# Hypothesis about Transdifferentiation As Backbone of Malignancy

**DOI:** 10.3389/fonc.2017.00126

**Published:** 2017-06-19

**Authors:** Jean Piechowski

**Affiliations:** ^1^Physician-Radiotoxicologist, Paris, France

**Keywords:** cancer, epigenetics, transdifferentiation, trophoblast, germ cells, stem-like cells

## Abstract

**Background:**

Cancer is mainly watched through the prism of random mutations and related corruption of signaling pathways. However, it would seem puzzling to explain the tumor organization, pugnacity and steady evolution of the tumorous disease and, moreover, a systematic ascendancy over the healthy tissues, only through stochastic genomic alterations.

**Malignancy specific properties:**

Considering the core characteristics of cancer cells, it appears that two major sets of properties are emerging, corresponding to well-identified physiological phenotypes, i.e., (1) the trophoblastic logistical functions for cell survival, protection, expansion, migration, and host-tissue conditioning for angiogenesis and immune tolerance and (2) the sexual functions for genome maintenance. To explain the resurgence of these trophoblastic and sexual phenotypes, a particular cell reprogramming, to be called “malignant transdifferentiation” in view of its key role in the precancer-to-cancer shift, appears to be a convincing hypothesis.

**Perspectives:**

The concept of malignant transdifferentiation, in addition to oncogenic mutations, would determine a more rational approach of oncogenesis and would open so far unexplored ways of therapeutic actions. Indeed, the trophoblastic phenotype would be a good candidate for therapeutic purposes because, on the one hand, it covers numerous properties that all are vital for the tumor, and on the other hand, it can be targeted with potentially no risk of affecting the healthy tissues as it is not expressed there after birth.

## Introduction

In spite of exhaustive description of cancer properties (1) and tracking of oncogenic and contingent mutations (2), it is still difficult to explain the singular evolution of the malignant process. Its main characteristics are in every case similar, systematically involving an endless clonal expansion together with a specific host reaction. It would be quite hard to explain such an uniqueness should cell malignancy be watched exclusively through the prism of a variable spectrum of random mutations and related cell-signaling corruption. Indeed, cells distressed by oncogenes or any intensive pro-growth constraints, and notably in case of a poor microenvironment, would develop an adaptive strategy to grow and prosper, consisting in resurgence of a well-defined set of dormant vital properties and logistical means. The core properties of cancer cells are not newly created properties that would appear in the course of the tumorous disease. They are physiological, normally silent properties, but here aberrantly expressed as a constitutive set of functions whose coordinating determines synergistic mechanisms of cell survival and expanding. The tight connection between the cancer cells and the host tissues, as manifested by the pro-tumor conditioning of the latter, is an integral part of the process. Thus, besides the abnormal cell behavior directly resulting from the impact of the various random mutations on cell-signaling pathways, a wide range of reprogrammed functions participates in completion of the oncogenic process. We will analyze the issue raised by the putative mechanism of resurgence of these functions, draw out the rationale for leading the cell to make them operational, and consider potential therapeutic application.

## Malignant Transdifferentiation

Transdifferentiation is an epigenetic process consisting in acquisition, by a cell of a given type, of the properties and characters of another cell type, in place of its own phenotype. Natural spontaneous transdifferentiation is a rare event, observed in some very specific cases, mainly relating to tissue regeneration (3–5). From a strictly theoretical point of view, it is possible to state that a given phenotype may be modulated toward—or replaced by—any other one, as this is basically a matter of gene expression. Experimentally induced alteration of differentiation may occur either passing *via* a clear phenotype resetting, i.e., a totipotent or pluripotent state, or not. Transdifferentiation refers to the latter option (6). Efficiency and kinetics are rather uneven. Cell plasticity, an epigenetic status endowing the cell with high cell identity flexibility, boosts the initial steps of the process. Increasing it through a transient cell preprocessing with stemness transcription factors—the same as those used to induce pluripotency—enlarges the spectrum of achievable cell lineages (7). As regards the epigenetic requirements for the differentiation switch, numerous *in vitro* studies have identified various cocktails of transcription factors to be used according to the starting and the desired final cell types (4). Ability to drive transdifferentiation is not limited to transcription factors as non-coding RNAs can promote it as well (8). The culture medium has an impact in terms of both phenotypic fate and efficiency. Transdifferentiation is now currently feasible *in vivo* (9).

**Figure 1:**
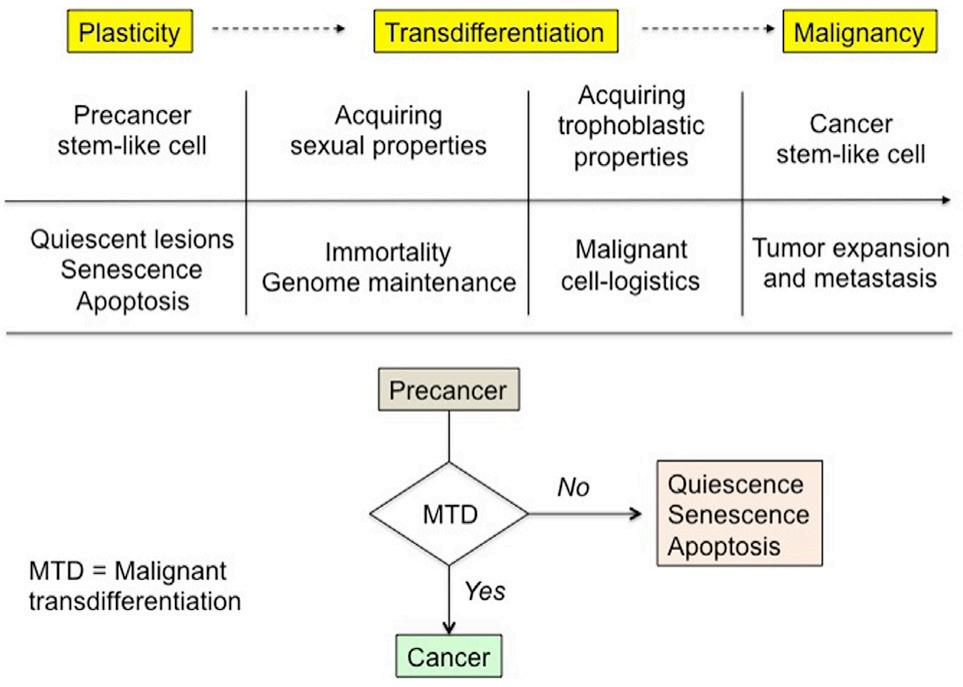
Malignant transdifferentiation and rationale of precancer-to-cancer transition. Physiological trophoblastic and sexual functions, strictly dormant in normal somatic cells, are supposed to be unlocked in cancer cells in order to fulfill the unusual both vital and expanding needs of the latter. The corresponding phenotypes are in some ways “Janus-faced” entities. For instance, the broadly similar embryo and cancer tissue needs are met through implementing the trophoblastic highly pro-survival properties and functions operating during embryo implantation. The triggering and the molecular basis of the process remain to be deciphered. In the absence of malignant transdifferentiation, premalignant lesions either slumber, remaining quiescent, or wither through senescence and apoptosis.

We hypothesize that cancer cells have vital and expanding needs they cannot meet through usual physiological means. This growth crisis would trigger an adaptive yet aberrant resurgence of highly efficient pro-survival phenotypes that join the primordial phenotype, thus forming a malignant hybrid phenotype. Indeed, besides their own original phenotype, cancer cells seem to achieve a particular transdifferentiation, acquiring trophoblastic and sexual properties—described in the next sections—whose pooling will determine their survival and active malignancy (Figure 1). The following fundamental fact implicitly underlies the proposed hypothesis. Trophoblastic and sexual genetic programs are present in the genome of any somatic cell of an individual but their expression is tightly blocked at every level from transcription to posttranslation. A clear proof of that, concerning for instance the trophoblastic program, is the reproductive cloning through somatic cell nuclear transfer, which leads to all the intra- and extra-embryonic structures, and in particular the placenta. The probability of completion of malignant transdifferentiation is supposed to be infinitely small as compared to the rate of potentially oncogenic mutations (10–13). This matches the fact that the cancer rate in the population would otherwise be disproportionate to the observed one.

Plasticity and related transdifferentiation potential represent a typical feature of stem cells, and in particular of tumor stem-like cells. Various well-identified conditions and factors commonly operating in cancer promote this epigenetic property:

- Stemness transcription factors, e.g., Oct4, Sox2, Nanog, Klf4, c-Myc, etc., sustain stem cell undifferentiated state and induce pluripotency (6, 14);
- Proteins of the PcG (polycomb) and TrxG (trithorax) groups involved in epigenetic modulation are pro-relaxing (TrxG) or anti-relaxing (PcG) on the epigenetic state (15);
- Genes controlling genome integrity, especially *TP53*, have a restrictive action on cell plasticity, i.e., promote epigenome stability, which could partly explain their tumor suppressor property (16). Transition of a somatic cell from a differentiated state toward a pluripotent state is impeded by p53, whereas the yield of this transition increases significantly when p53 is repressed (17). Therefore, mutation with loss of function of *TP53*, a frequently occurring genetic defect in cancer, most likely fosters cell plasticity, thus paving the way for transdifferentiation. Some other either inherited or acquired genetic and chromatin defects presumably exist that may have the same consequence;
- Hypoxia-inducible factor (HIF) seems to promote cell pluripotent stem state (18, 19);
- Autophagy supports stemness in defective cell conditions (20).

Conversely, rigorous plasticity control, with efficient locking of the master genes governing the aforesaid sexual and trophoblastic phenotypes, could be the prime reason why oncogene-mutated cells and precancerous foci may either remain quiescent with no transition to active malignancy or are prone to senescence and apoptosis, being understood that tumor suppressor genes that control genome integrity, mainly the p53 network, interfere in this process (21).

## Trophoblastic-Like Transdifferentiation

Trophoblast is the extra-embryonic structure that will form the placenta. Young embryo is quite vulnerable and the trophoblastic program is suitable to meet the needs of the early steps of development, in spite of a semi-foreign immunological status and of hard physiological post-nidation conditions like hypoxia, and even in adverse situations as for instance ectopic pregnancies or embryo major genetic defects. Since the trophoblastic functions are so efficient for cell survival and expansion, they would make the cancer cells very robust and able to overcome healthy tissues. Indeed, cancer cells present strong similarities with the physiological early trophoblast. More specifically, cancer and extravillous cytotrophoblast cells share a wide set of common logistical properties relating to cell protection, proliferation, and invasiveness, implemented by the same factors. These numerous properties and functions, largely documented in detail (22–25), are summarized with several additional points and references in the below list:

- Epithelial–mesenchymal transition (EMT) and mesenchymal mode of cell migration during implantation and host-tissue invasion. Leukemia inhibitory factor (LIF) and cytokines of the transforming growth factor β (TGF-β) superfamily promote the process that furthermore requires both secretion of proteolytic enzymes like matrix metalloproteinases and expression of pro-migration cadherins and integrins. The urokinase-type plasminogen activator (uPA)−urokinase receptor (uPAR) axis induces plasmin release and protease activation in the vicinity of the migrating cells. In addition to proteolysis, the uPAR regulates cell signaling and adhesion in a way that promotes migration. Its action is determined by both uPA-dependent and uPA-independent mechanisms (26, 27). Cancer cells may yet use—or switch to—the more trivial and faster amoeboid—in place of mesenchymal—mode of migration (28). Furthermore, to widen the scope of this topic, it seems relevant to draw a parallel between the migration and metastasis of cancer cells in the host organism, and the fetomaternal microchimerism involving *inter alia* dissemination and retention of trophoblastic cells in the pregnant woman (29);
- Implementing stroma neovascularization through vascular endothelial growth factor (VEGF) and placental growth factor (PGF)-induced angiogenesis combined with vascular mimicry;
- Metabolic and cell energy special features. They mainly consist in HIF-related anabolic–glycolytic metabolism characterized by aerobic glycolysis and an enzymatic pattern favoring cell component synthesis, BB-creatine kinase as surrogate energy vector, and flexible use of autophagy to survive critical situations;
- High expression level of the scavenger receptor class B, type 1, mediating cell selective uptake of cholesterol esters from high-density lipoprotein (30, 31);
- Apoptosis inhibitors, various factors specifically targeting and weakening the host immune reaction against the colonizing tissue, effective agents of xenobiotics processing, all of which being complementary key actors of cell protection. In spite of not being strictly speaking a foreign tissue relatively to the host organism, cancer tissue benefits from the trophoblastic immune tolerance mechanisms that physiologically protect embryo, an actual foreign being, against the maternal immunological reaction;
- Autotaxin (ATX)–lysophosphatidic acid (LPA)–lysophosphatidic acid receptors (LPARs) axis. The extracellular LPA is produced by action of the ectoenzyme ATX, a secreted lysophospholipase D known as “autocrine motility factor,” on circulating lysophosphatidylcholine. It is degraded by lipid phosphate phosphatases. LPA is a potent multifunctional mediator that intervenes in both cell fate and functioning, and production of chemokines, through activating a series of specific G protein-coupled receptors (LPARs), see, for instance, the role of Rac1 in host-tissue conditioning later in this section. The ATX–LPA–LPARs signaling axis has emerged as an important regulation player in both trophoblast and tumors (32, 33). It contributes to cell growth, motility and invasiveness, and angiogenesis and has pro-survival properties;
- Common growth factors and growth factor receptors, autocrine loops;
- Similar DNA methylation profile fostering oncogene expression and tumor suppressor gene repression;
- Synthesis of non-coding RNAs that promote cell growth, migration, and invasiveness, e.g., the oncomir miRNA-21 (34, 35) and the long ncRNA metastasis associated lung adenocarcinoma transcript-1 (36, 37). They modulate expression of a large number of genes. Knowledge of their regulation, efficiency, and targets is still partial;
- Syncytin-related cell fusion. Multinuclear cells are present in trophoblast as syncytiotrophoblast and cytotrophoblast giant cells, and they may be observed in cancer as giant cells;
- Gap junctional intercellular communication. Connexins, tightly regulated short-lived transmembrane proteins, form channels for cytoplasmic connecting between adjacent cells. They occur in all tissues, except in the skeletal muscles, and are strongly active in the trophoblast. In cancer tissue, they link tumor cells to tumor or normal cells. Their importance and role in the cancer process still remain to be fully assessed. Besides the issue of their alterations, they seem to have an ambivalent role, impeding early stages and conversely promoting late stages of cancer progression (38–40);
- Secretion of the human chorionic gonadotropin hormone (partial secretion of the β-chain in advanced stages of tumors);
- Similar oncofetal proteins as cell biomarkers.

A critical issue is the conditioning of the host-tissue stroma. Trophoblast or tumor invasion create conditions that can be broadly likened to tissue wounding and hence are prone to trigger the tissue-repair machinery. Many growth factors are potentially involved, together with inflammation signaling pathways, e.g., the toll-like receptor–myeloid differentiation primary-response protein 88 (MyD88) axis that is activated through tissue and cell debris (41, 42). This signaling pathway, with MyD88 as an adaptor molecule, functions in the control of inflammation both in tissue repair and in host innate immune defense from infection. Proper course of pregnancy requires that the decidua becomes an immunologically distinctive site that allows the invasion and growth of the semi-allogeneic trophoblast, while paradoxically maintaining host defense against an array of microbial pathogens. This immunological duality could also apply to the tumorous tissue. More specifically, malignant stroma designing takes the form of a set of singular properties like notably neovascularization, secretion by stromal cells of a collection of synergistic factors supporting cancer cells (43), and formation of a myofibroblastic microenvironment with production of pro-tumor extracellular matrix by cancer-associated fibroblasts (CAFs) (44). The pro-invasive LIF and cytokines of the TGF-β superfamily regulate the tumor stroma structuring. CAFs are mesenchyme-related, TGF-β responding cells like resident fibroblasts, fibroblast-like leukocytes, stellate cells (45), and decidual stromal cells (46, 47). Depending on their involvement in support of various physiological or pathological processes like wound healing, pregnancy decidua conditioning, fibrogenesis, or cancer tissue growth, all these cells express α-smooth muscle actin, a marker of fibrogenic myofibroblastic phenotype. Rac1 GTPase, a RAS superfamily/Rho-protein family member and pleiotropic regulator of many cellular processes, acts as a booster of stromal cell motility in the operations conditioning the decidua for embryo implantation and the tumor microenvironment for cancer progression (48–50). Among the chemotactic cytokines, interaction between trophoblast and decidua cells or between cancer cells and their stromal neighbors involves more specifically the CXC chemokine subfamily where C is an N-terminal cysteine and X is an intercalated amino acid. For instance, the CXCL12 (ligand)–CXCR4 (G protein-linked transmembrane cell receptor) axis sustains the invasive process (51, 52). Besides the concept of cancer stem-like cell niches, a linked topic is the primary tumor involvement in preparing distant premetastatic niches. Released exosomes along with secretions rich in factors of the VEGF family, including the PGF, trigger activation and recruitment of vascular endothelial growth factor receptor 1-expressing myeloid cells that will locally develop favorable places for metastatic colonizing (53). Trophoblast as well as cancer cells interact with the host organism to promote the recruitment of myeloid-derived suppressor cells that are effectors of immune tolerance (54, 55).

To explain the common features shared by the trophoblast and cancer cells regarding their functional connection with the host organism, a credible assumption may be that the pro-tumor reprogramming of local and systemic non-cancer cells is induced by—and linked to—the trophoblastic-like transdifferentiation of cancer cells. The specific feature of trophoblastic and malignant cells is their potent expanding ability based on two key mechanisms:

- The use, respectively physiological and pathological, of a common enzymatic machinery to lyse healthy normal tissues in order to invade them;
- A positive feedback loop between the colonizing cells and the host tissues, enabling these cells to manage a continuous increase of pro-survival places.

Accordingly, it is commonly stated that trophoblastic cells transiently adopt a tumor-like phenotype. However, it does not seem to be really logical considering that a physiological process systematically includes a tumor-like step. Conversely, what seems to better make sense is to consider that the trophoblastic properties are constitutively reprogrammed in cancer cells, with the clear restrictive observation that their expression is neither time nor space limited in this case. Indeed, the trophoblastic logistical support is essential, being continuously required for cell survival and expanding. As this takes priority over any functional setting on standby, the physiological regulation implemented during pregnancy is not operating here.

**Figure 2:**
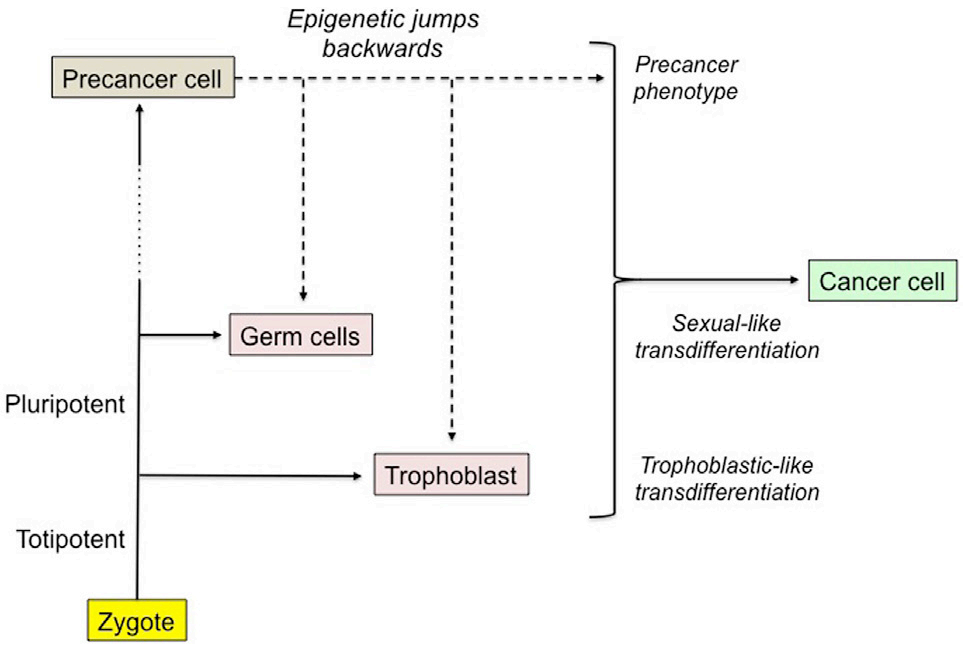
The epigenetic jumps backward leading to the malignant phenotype. Any cell of an individual derives downstream from the zygote after a lot of cell divisions, a very long epigenetic trip and possibly various mutations, certain of them promoting a precancerous state. Hence, the genes present in the zygote and in each of the subsequent cells are the same, yet with various structural and/or epigenetic alterations. We hypothesize that a precancer cell becomes malignant after it achieves epigenetic jumps backward leading to the reprogramming of normally turned off sexual and trophoblastic master genes. Malignant cell would be a phenotypic hybrid made of the primordial precancer cell supplemented by sexual-like and trophoblastic-like transdifferentiations. The probability of the process is infinitesimal and the mechanism still remains to be explored. The expression of oncofetal biomarkers could be a related collateral effect.

Malignant trophoblastic-like transdifferentiation would correspond to a considerable epigenetic jump backward as trophoblast derives from totipotent cells, soon after the zygote starts to develop (Figure 2). This rejuvenation provides the cell with both invasive properties and high functional adaptability to the host tissues. Exploring the epigenetic reprogramming of the trophoblastic master genes as a major cause of malignancy should be a key challenge.

## Sexual-Like Transdifferentiation

Sexual cells are endowed with sophisticated enzymatic machinery for genome maintenance, restructuring, and reparation. As cancer cells are facing genome defects and instability concurrently with continuous multiplication, they have a compelling need of efficient means for genome maintenance. They clearly re-expressed certain genome supporting functions typical of germinal cells (56).

As cell immortality that depends on telomere regeneration is a core condition for unlimited clonal expansion, most of cancer cells (about 85–90%) have an active reprogrammed telomerase. Although telomerase is commonly spontaneously expressed in somatic stem cells, it may also be a resurgence of the germinal phenotype. In Hayflick-type experiments consisting in forced, unlimited continuous cell proliferation, the probability of escaping the fatal so-called “crisis” thanks to telomerase re-expression is of the order of 10^−6^ or less (57). Another hallmark of malignant cells as well as of normal placenta, unquestionably of sexual origin, consists of expression of cancer testis antigens (CTAs) (58, 59). Some of the related genes may be epi-drivers, i.e., pro-cancer genes activated through an epigenetic process (60). Interestingly, this could be part of a sexual-like transdifferentiation. Hundreds of CTAs have been identified but the actual function of a large proportion of them still remain to be clearly and unequivocally established. Nevertheless, various putative pro-tumor properties are commonly attributed to them, having essentially to do with prevention of differentiation, and cell survival and growth. More specifically, certain CTAs correspond to proteins involved in genome maintenance as for instance support to mitotic fidelity (61) and proper achievement of homologous recombination during meiosis (62–65). Besides their physiological role in meiosis, the latter also participate in the alternative lengthening of telomeres in cases where telomerase remains inactive, which occurs in about 10–15% of tumors (66, 67). In addition, a plagiarism of the meiotic interchromosomal genetic mixing may lead to various iterative chromosome rearrangements resulting in an increase of the tumor heterogeneity (68).

## Hypothesis about a Malignant Triumvirate and Research Perspectives

**Figure 3:**
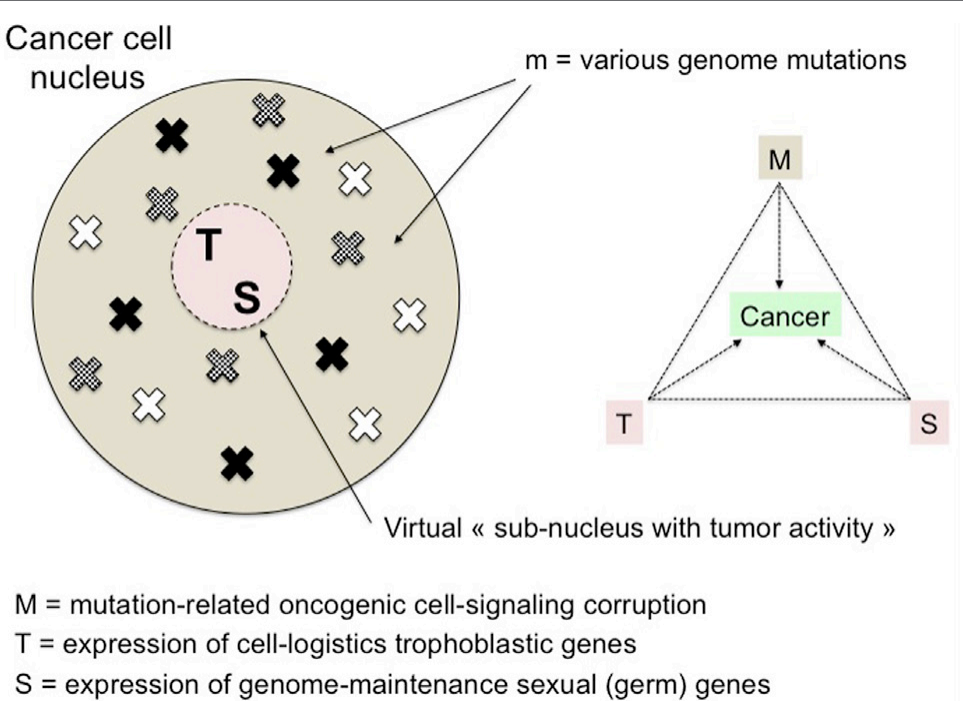
The malignant triumvirate. Malignancy is supposed to be the result of the convergence of three anomalies: mutations (and related signaling corruption), and resurgent expression of trophoblastic and sexual master genes. M is variable and determines the multiplicity of cancer processes whereas T and S are fixed actors of the disease. Trophoblastic and sexual genes may be considered as components of a virtual “malignant subnucleus” tightly neutralized in normal cells. Its infinitely low-probability—and so far unexplored—more or less progressive “reactivation” is supposed to be the optimal way for cancer cells to acquire the necessary multi-function support to survive and grow.

Mutations and cell-signaling corruption coupled with the putative trophoblastic and sexual transdifferentiations could be considered as the malignant base of cancer cells (Figure 3). These transdifferentiations alter the cell properties and the stroma, in a way that favors the worst aspects of malignancy, i.e., invasiveness, resistance to host defenses and therapy, and relapses. Dormancy and high resistance to stress and xenobiotics are well-known features of stem cells. The resilience and resistance of cancer stem-like cells would certainly be boosted by the reprogrammed trophoblastic properties, as trophoblast derives directly from totipotent cells, i.e., the cells with the highest stem potential just after the zygote. It is more difficult for a cell to become totipotent than pluripotent, i.e., respectively acquiring potentiality of producing extra-embryonic structures or not, so that sexual reprogramming, notably cell immortality, would certainly occur before trophoblastic reprogramming in the course of the cancer process (Figures 1 and 2). Epigenetic control of the trophoblastic and sexual master genes is probably the key point regarding the demarcation between precancerous lesions and malignancy. This could be brought closer to and indeed be a rationale of the basic concept of tumor promotion, thus raising new issues about the related factors and mechanisms.

In the proposed hypothesis, it is important to notice that the trophoblastic and sexual properties, which provide, respectively, migration capability and potential immortality, are simply added to the original set of cell properties. Thus, the cells maintain their own tissue-specific characteristics, cell-cycle progression and differentiation trajectories—potentially altered by oncogenic mutations—but they are now endowed with pro-survival and pro-invasive attributes. The additional properties are *de novo* exclusively acquired by stem-like cells, as these cells are the only ones able to achieve transdifferentiation due to their phenotypic plasticity. Considering a model of tumor development based on stem-like cells producing an increasingly differentiated progeny with loss of self-renewal and decrease of proliferative potential, it appears that only the stem-like cells would be able to form metastasis. Indeed, even if the differentiated cells may eventually migrate due to the trophoblastic properties they inherited from the parent cells, they are by themselves unable to create lasting metastatic sites since their progeny gradually becomes a postmitotic population ultimately doomed to disappear, albeit possibly over long periods because of the multifaceted deregulation of cell growth, including *inter alia* the loss of physiological barriers like the telomere length. Therefore, it is most likely the transdifferentiated stem-like cells expressing trophoblastic and sexual properties that are the actual core actors of tumor spreading and persistence, and thus of cancer malignancy. This assertion is reinforced by the fact that the population of stem-like cells progressively increases due to symmetric divisions occurring in addition to the more usual asymmetric divisions.

The behavior of the host tissue toward the tumor seems to be ambivalent if we consider for instance the opposite effects of immune assault and tumor neovascularization on the tumor development. In fact, we hypothesize that the pro-survival trophoblastic phenotype works as during embryo implantation, i.e., it counteracts the host antitumor actions, e.g., through muting immune reaction and pro-apoptosis mechanisms, while it forces the host tissue to support the tumor, e.g., through promoting angiogenesis. Furthermore, a gradual loss of differentiation is commonly observed in the population of cancer cells. Two complementary mechanisms are credible, i.e., defective pro-differentiation pathways in the cells and lack of pro-differentiation stimuli from the microenvironment. Finally, as the undifferentiated status is in progression, the trophoblastic phenotype becomes an actual leading cell driver along with the deregulated cell growth and the resulting aberrant proliferation. All this makes the malignant disease more and more aggressive and expanding. The immortality related to the constitutive telomere maintenance—a component of the sexual phenotype—determines the endless nature of the process and thus indirectly promotes the multifocal dissemination of the cancer tissue in the host organism.

Cancer cells are faced the challenge of finding an optimal editing of the epigenome to meet multicomponent vital and growth needs that may change over time according to the evolution of both mutations and microenvironment. Thus, tumors do likely present a dual heterogeneity, one structural according to the type and the number of mutations, and the other functional relating to the quality and the extent of the trophoblastic and sexual transdifferentiations, both of them evolving over time. The huge diversity of the structural defects sharply contrasts with the broadly invariable sequence of the malignancy clinical events. This could reflect the contrast between the high variability of the spectrum of the genomic alterations and the well-defined characteristics of the putative transdifferentiations underlying the malignant process.

In practice, a pivotal objective should be demonstrating that the trophoblastic properties observed in cancer cells result from expression of the master genes governing the extravillous cytotrophoblast cells, these being the actually proliferative and invasive trophoblastic cells (25, 69). Further assessment of the pro-trophoblast biased cell pattern is needed. This implies additional investigation about aberrant expression of extravillous trophoblast phenotypic attributes in cancer cells, regarding various items: biomarker profiles (70), transcriptome (71, 72), methylome (73), signaling pathways and related transcription factors (74), metabolic pattern (75, 76), and interactions with normal somatic cells, e.g., through adapted coculture systems (77, 78). An essential issue that remains to be addressed is the possible analogy between the epigenetic control of the physiological trophoblastic differentiation and that of the trophoblastic reprogramming of cancer cells. This would require exploring two complementary epigenomics-related domains, i.e., (1) upstream, characterize the mutations and epigenetic shifting that most likely foster abnormal cell plasticity and enable subsequent unrestricted expression of the trophoblastic master genes. This very rarely achieved and as yet unexplored putative process is supposed to be the pro-survival response to cancer-related stresses, i.e., intrinsic cell growth crisis and extrinsic impediments like poor microenvironment, unfit blood supply, hypoxia, limited space, persistent inflammation, immune reaction, and fibrosis. It allows cell escaping senescence and apoptosis through acquiring essential support for growth, progeny expanding, and protection, and (2) downstream, identify the inventory of the master transcription factors able to induce transdifferention toward the trophoblastic phenotype, by extending available data (79–87). Such information would be useful for any future research on the cell-signaling axes shared by trophoblastic and cancer cells. Transfection of cells exposed to carcinogens with the genes coding for these transcription factors—or conversely their silencing mediated by non-coding RNAs in cancer cells—would allow us to assess the trophoblastic impact on the malignant transformation.

However, interpretation of the specific role of transcription factors in the tumor processes is not so obvious as one might surmise *prima facie*, this being due to (1) their common involvement in several signaling pathways that could be in conflict regarding the tumor evolution, (2) the impact of interfering regulatory circuitries, and (3) unexpected alteration or dysfunction of certain components of the signaling network. Moreover, the knowledge of the genetic and epigenetic organization of trophoblastic cells is so far not enough developed and precise to be used with confidence and to avoid any misinterpretation. Data on trophoblastic transcription factors and related putative phenotypic impact are partial, often arduous to interpret, and present significant inter-species variation. Up to now, there is no comprehensive logic diagram, not even an advanced outline of how really the system functions. Therefore, use of conventional biological means as for instance evaluation of cell behavior, biomarkers, cell receptors, metabolic patterns, secreted factors, etc., may possibly be more appropriate and efficient to understand the underlying mechanisms of the malignant process. Such facts were presented here and striking evidence appeared as to the trophoblast and cancer similarities, in a lot of independent domains. Further work is now needed, strictly and specifically devoted to the comparison of the transcriptional features between trophoblastic and cancer cells, searching for putative matching. This could be the next step after the hypothesis of malignant transdifferentiation addressed in this paper.

**Figure 4:**
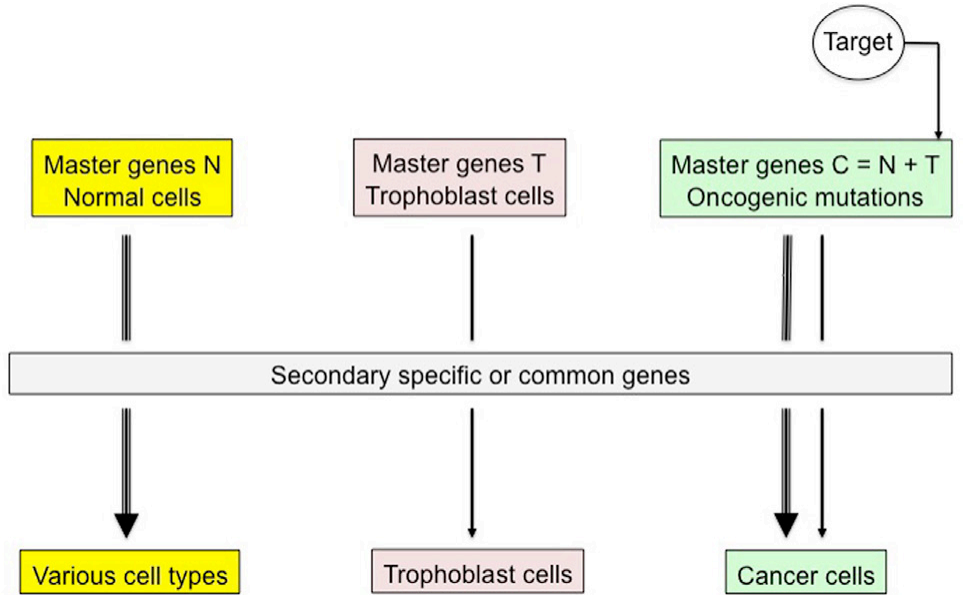
Specific therapeutic targeting of trophoblastic master genes. Trophoblastic transdifferentiation is supposed to promote active malignancy in mutated, precancerous cells. Blocking expression of trophoblastic master genes or of related transcription factors and cell biomarkers would exclusively have an impact on cancer cells, thus jeopardizing the tumor with *a priori* no consequence on normal somatic cells where trophoblastic genes are tightly locked. For instance, immunological targeting of tumor trophoblastic components could be a good therapeutic option.

The reprogrammed trophoblastic phenotype would be a particularly good candidate for therapeutic projects because of two favorable key characteristics: (1) it does cover numerous properties that are essential in supporting the tumor growth, protection and expansion, and (2) its targeting would potentially be with no risk of adverse effects in the healthy tissues as it is not expressed there after birth (Figure 4). The latter condition does not apply to the germinal phenotype, thus making this phenotype rather not relevant for therapy purposes. The current specific therapies are either targeting the pro-growth receptors and kinases of malignant cells, or inhibiting angiogenesis and the checkpoints that limit immune response intensity. Besides becoming increasingly complex, they *de facto* interfere with the normal tissues and have adverse effects, altering the quality of life with possibly serious consequences. This would not be the case of anti-trophoblast-based therapies exclusively hampering expression of the trophoblastic genes or destabilizing their specific signaling pathways and cell biomarkers (25). Only the tumor logistics, as notably the aberrant pro-invasive, anti-apoptosis, pro-angiogenesis, and immune-muting properties would be impacted, making cancer cells severely declining, with *a priori* no impairment of the healthy tissues, in particular with regard to tissue renewal, vascular integrity, and immune status.

## Conclusion

The basic assumption is that the pugnacity of cancer, notably cell immortality and invasiveness, cannot be the outcome of just an accumulation of random mutations. In fact, potent mechanisms supporting tissue growth and cell protection are involved. They correspond to certain properties transitorily expressed during embryogenesis and then normally kept strictly dormant, yet resurfacing here as constitutive functions. Many of cardinal features of malignancy, in particular the logistics of cell functioning and genome maintenance, match the trophoblastic and sexual phenotypes. We hypothesize that their resurgence is not a consequence of a random process but rather the result of an uncommon epigenetic cell redesigning to be called malignant transdifferentiation. Accordingly, cancer cells may be considered as phenotypic hybrids comprising the mutation-altered primary, the trophoblastic and the sexual phenotypes. This concept, which could be considered as the cornerstone of the precancer-to-cancer progression, should be explored going further in the analysis of its mechanisms, especially concerning the trophoblastic component because of potential therapeutic development. Indeed, trophoblastic properties are crucial for the tumor while not expressed in normal tissues after birth, thus appearing *a priori* as an ideal target. Finally, it would be of great interest bringing closer research on biology of cancer and embryology with a focus on the properties of trophoblast, searching for its Achilles’ heels.

## Author Contributions

The author confirms being the sole contributor of this work and approved it for publication.

## Conflict of Interest Statement

The author declares that the research was conducted in the absence of any commercial or financial relationships that could be construed as a potential conflict of interest.

## Abbreviations

ALT, alternative lengthening of telomeres; ATX, autotaxin; CAF, cancer-associated fibroblast; CTAs, cancer testis antigens; EMT, epithelial–mesenchymal transition; GJIC, gap junctional intercellular communication; HDL, high-density lipoprotein; HIF, hypoxia-inducible factor; LIF, leukemia inhibitory factor; LPA, lysophosphatidic acid; LPARs, lysophosphatidic acid receptors; LPPs, lipid phosphate phosphatases; MALAT-1, metastasis associated lung adenocarcinoma transcript-1; MDSC, myeloid-derived suppressor cells; MyD88, myeloid differentiation primary-response protein 88; PcG, polycomb group; PGF, placental growth factor; SR-B1, scavenger receptor class B, type 1; TGF-β, transforming growth factor β; TLR, toll-like receptor; TrxG, trithorax group; uPA, urokinase-type plasminogen activator; uPAR, urokinase receptor; VEGF, vascular endothelial growth factor; VEGFR1, vascular endothelial growth factor receptor 1.
